# Successful Catheter Ablation of Two Macro-reentrant Atrial Tachycardias in a Patient with Congenitally Corrected Transposition of the Great Arteries: A Case Report

**DOI:** 10.19102/icrm.2020.111005

**Published:** 2020-10-15

**Authors:** James Pennoyer, Michael Bykhovsky, Daniel Sohinki, Rachel Mallard, Adam E. Berman

**Affiliations:** ^1^Division of Adult Cardiology, Medical College of Georgia, Augusta, GA, USA; ^2^Division of Pediatric Cardiology, Medical College of Georgia, Augusta, GA, USA

**Keywords:** Atrial tachycardia, catheter ablation, congenitally-corrected transposition of the great arteries

## Abstract

Adults with congenital heart disease represent a complex and growing patient population. By virtue of their variant anatomy and the complex surgical repair often required in infancy, these patients are at risk of developing unique atrial and ventricular arrhythmias throughout their lifetimes. Electrophysiologists involved in the care of these patients should have a detailed understanding of their underlying anatomy and any prior surgical procedures to guide procedural planning and should have knowledge of the range of possible arrhythmia mechanisms that may differ from patients without structural heart disease. Despite this complexity, standard mapping techniques and electrophysiologic maneuvers may still be used to elucidate arrhythmia mechanisms, map tachycardia circuits, and guide catheter ablation. We report a case of two different macroreentrant right atrial tachycardias that were successfully ablated in a patient with congenitally-corrected transposition of the great arteries.

## Introduction

Congenitally corrected transposition of the great arteries (cc-TGA) is a rare complex congenital heart condition with a prevalence of 1 in 33,000 live births and that is characterized by atrioventricular (AV) and ventriculoarterial (VA) discordance. As the morphologic right ventricle (RV) supplies oxygenated blood to the systemic circulation and the morphologic left ventricle (LV) supplies deoxygenated blood to the pulmonary circulation, patients are not typically cyanotic. However, cc-TGA is frequently associated with other congenital cardiac defects, including ventricular septal defects (VSDs) (60%–80%), pulmonary stenosis (30%–50%), and conduction system abnormalities (15%–50%).^1^ As a result of anatomic and physiologic sequelae, cc-TGA patients are at risk of developing AV block as well as a variety of complex atrial and ventricular arrhythmias.^2–4^ We discuss the case of a 15-year-old female with cc-TGA and a number of associated congenital cardiac malformations who presented with highly symptomatic supraventricular tachycardia (SVT) causing syncope manifesting as two different macro-reentrant right atrial (RA) tachycardias.

## Case report

**Figure 1:**
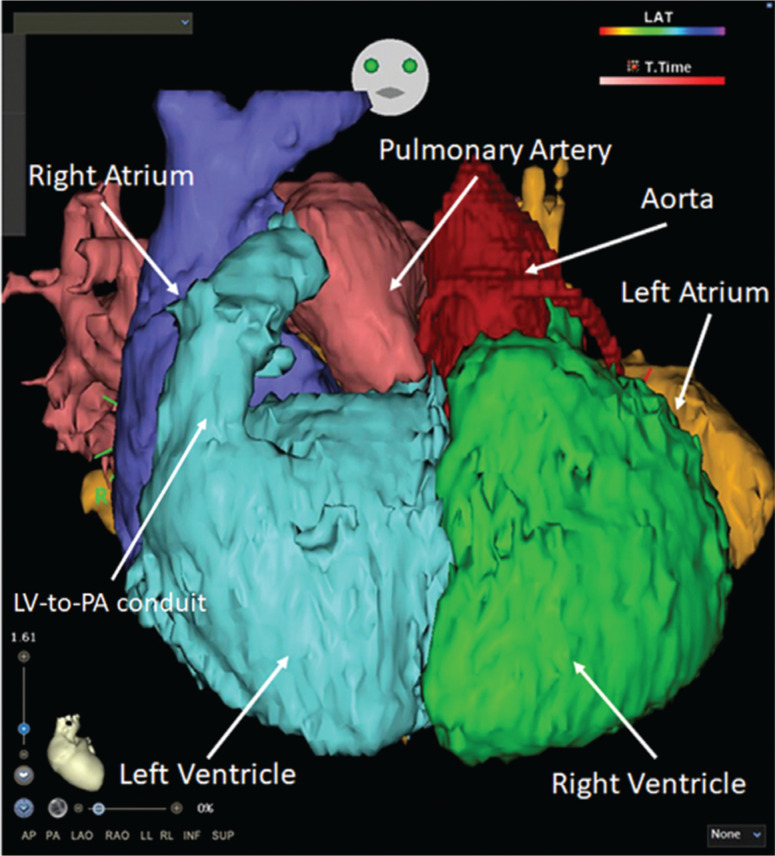
Patient anatomy: cardiac chambers and great vessels. Both AV and VA discordance are present in cc-TGA. Thus, a morphologic RA drains into a morphologic LV through a mitral valve, then ejects into the main PA. Similarly, a morphologic left atrium drains into a morphologic RV through a tricuspid valve, ejecting into the ascending aorta. Note the LV-to-PA conduit that was surgically constructed to palliate obstruction of the RV outflow tract. PA: pulmonary artery; RVOT: right ventricular outflow tract.

A 15-year-old female with a medical history of cc-TGA, perimembranous VSD status following surgical closure, severe tricuspid regurgitation status following right atriotomy and mechanical tricuspid valve replacement, and pulmonary valvar and subvalvar stenosis status following LV-to–pulmonary artery (PA) extracardiac conduit with subsequent thrombosis **(Figure 1)** was transferred to our hospital following an episode of syncope and tachycardia. On the day of her presentation, she reported showering and experiencing a sudden onset of chest pain, palpitations, shortness of breath, headaches, and vision changes. This was accompanied by syncope. Her family also reported an episode of near-syncope approximately two weeks before this event; at the time, it was attributed to summer heat and underhydration. At an outside facility, her heart rate was recorded as 238 bpm and an electrocardiogram (ECG) confirmed a diagnosis of SVT. A dose of atenolol 12.5 mg was given, which resulted in brief periods of sinus rhythm, followed by the resumption of sustained SVT. She was also given three boluses of adenosine (6 mg, 6 mg, and 12 mg) without tachycardia termination. She was finally given an intravenous dose of 5 mg metoprolol, after which point, she converted to sinus rhythm. She was then transferred to our pediatric intensive care unit (ICU).

On initial examination, she was afebrile and hemodynamically stable with a blood pressure of 113/65 mmHg and a heart rate of 120 bpm. Her cardiac examination revealed a resting tachycardia with a regular rhythm. She had a grade 3 harsh systolic ejection murmur at the left sternal border, normal mechanical valve click, and prominent apical heave. Her physical examination was otherwise unremarkable. Laboratory data on admission including serum chemistries, complete blood count, thyroid function studies, and cardiac biomarkers were unremarkable.

**Figure 2:**
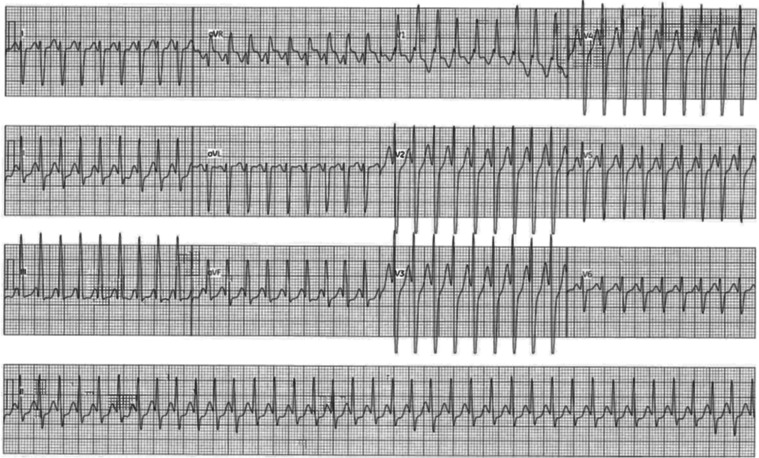
Presenting SVT. The standard 12 leads were recorded simultaneously. The lead II rhythm strip is a continuous recording.

A review of her initial ECG demonstrated suspected atrial tachycardia with 1:1 AV conduction and baseline right bundle branch block **(Figure 2)**. A transthoracic echocardiogram showed expected postsurgical changes related to her congenital heart disease (CHD) and a normally functioning tricuspid valve prosthesis. She also demonstrated underlying moderate systemic RV enlargement with RV systolic dysfunction.

**Figure 3:**
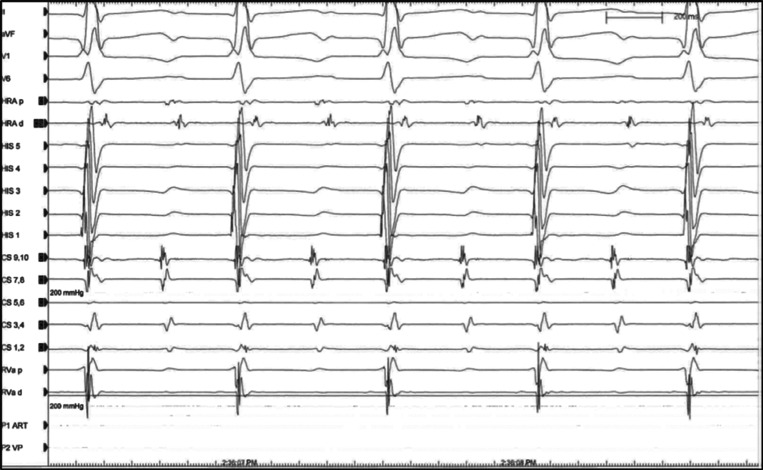
SVT1. SVT1 was noted to be a long-R–P tachycardia with 2:1 AV conduction, excluding orthodromic AV reentrant tachycardia and making AV-nodal reentrant tachycardia less likely, although 2:1 AV-nodal reentrant tachycardia with a lower common pathway block is not definitively excluded on this tracing. HRA: high right atrium (located in the RA appendage); RVa: right ventricular apex. CS 9,10 is located at the coronary sinus ostium. The His catheter is positioned in the basal right ventricle, inferior to the expected His bundle position.

Given her history of complex CHD and highly symptomatic SVT, she was referred for invasive electrophysiologic study. At baseline, she had no evidence of dual AV nodal physiology on either decremental atrial pacing or during the measurement of the antegrade AV-nodal effective refractory period. The location of the AV nodal complex was usually situated. Retrograde atrial activation was decremental and concentric. Burst pacing from the RA appendage induced SVT (with a tachycardia cycle length of 262 ms) **(Figure 3)**. Atrial and ventricular activations were dissociated during tachycardia, excluding orthodromic reciprocating tachycardia and making AV-node reentry less likely.

**Figure 4:**
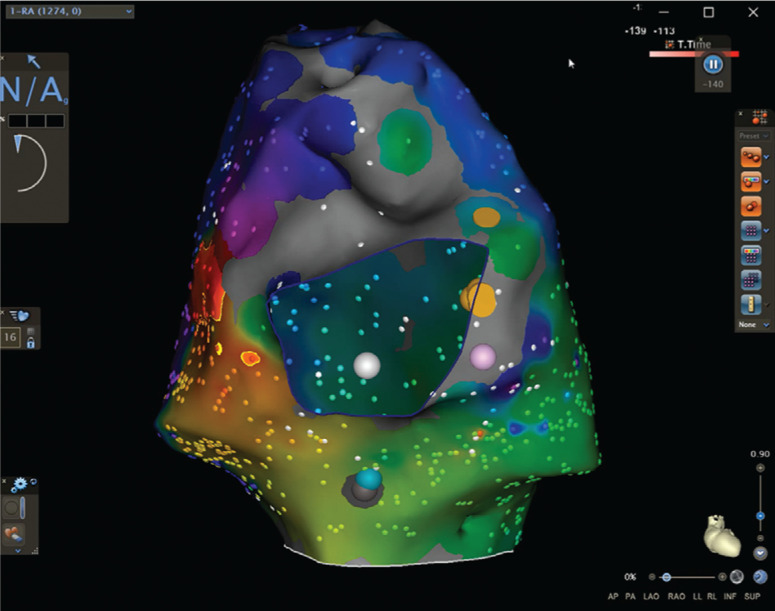
Activation map of SVT1. The image shows a left anterior oblique projection of the RA. The endocardial activation sequence is consistent with counterclockwise reentry around the mitral valve. Because of the presence of cc-TGA, the subeustachian isthmus is a cavomitral rather than cavotricuspid isthmus. Yellow tags: His recording locations; white, blue, and pink tags: locations of other attempts at entrainment.

**Figure 5:**
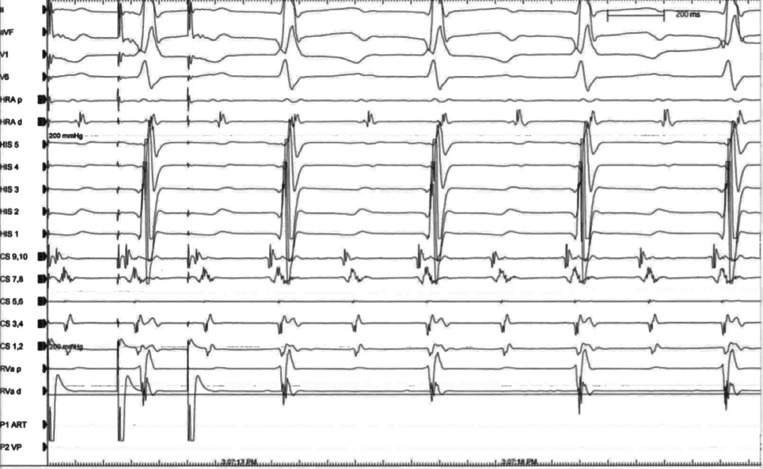
Entrainment of SVT1. Entrainment is performed with the pacing catheter positioned in the cavomitral isthmus. Note the downstream/upstream effect evident in this tracing as there is orthodromic capture of the high RA electrogram, identifying reentry as the tachycardia mechanism. The postpacing interval of the tachycardia cycle length was 0 ms in this location. CS: coronary sinus; HRA: high right atrium; RVa: right ventricular apex. The His catheter was located in the basal RV, inferior to the His recording location.

High-density mapping of the RA was performed during tachycardia with the PentaRay^®^ catheter (Biosense Webster, Diamond Bar, CA, USA) using the Carto^®^ 3 electroanatomic mapping system (Biosense Webster, Diamond Bar, CA, USA). Propagation mapping was consistent with counterclockwise cavomitral isthmus– dependent atrial flutter **(Figure 4)**. A ThermoCool SmartTouch^®^ ablation catheter (Biosense Webster, Diamond Bar, CA, USA) was positioned in the cavomitral isthmus and entrainment from this location yielded concealed fusion and a postpacing interval equal to the tachycardia cycle length **(Figure 5)**.

Ablation was performed during tachycardia in a slightly lateral location along the cavomitral isthmus to create a continuous lesion from the mitral annulus to the inferior vena cava. It was noted during catheter manipulation along this region that the curvature of the mitral annulus was demonstrably less convex than as is typically appreciated during cavotricuspid isthmus ablation. Tachycardia terminated during radiofrequency (RF) energy delivery, but pacing from the proximal coronary was suggestive of residual conduction through the isthmus. A series of consolidation lesions were delivered, yielding widely split double potentials, and bidirectional block was demonstrated using differential pacing. Repeat mapping was also performed during proximal coronary sinus (CS) pacing, which demonstrated “high-to-low” activation, consistent with block across the isthmus.

**Figure 6:**
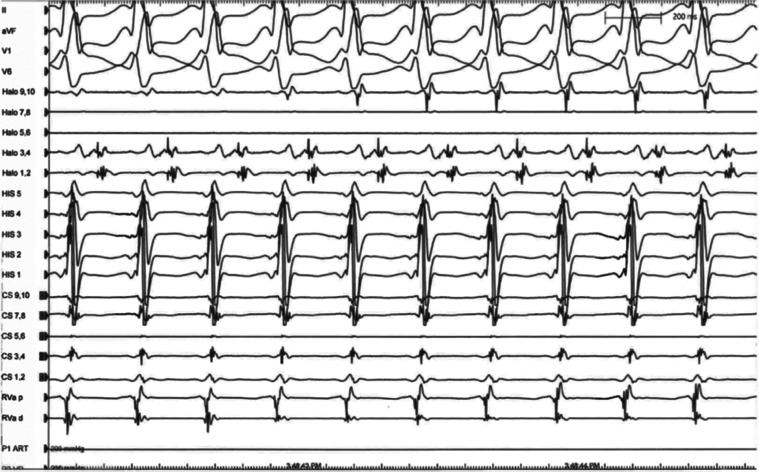
SVT2. Note the slightly different activation sequence in the CS recordings relative to that of SVT1. A halo catheter was placed prior to tachycardia mapping to further delineate the RA activation sequence.

**Figure 7:**
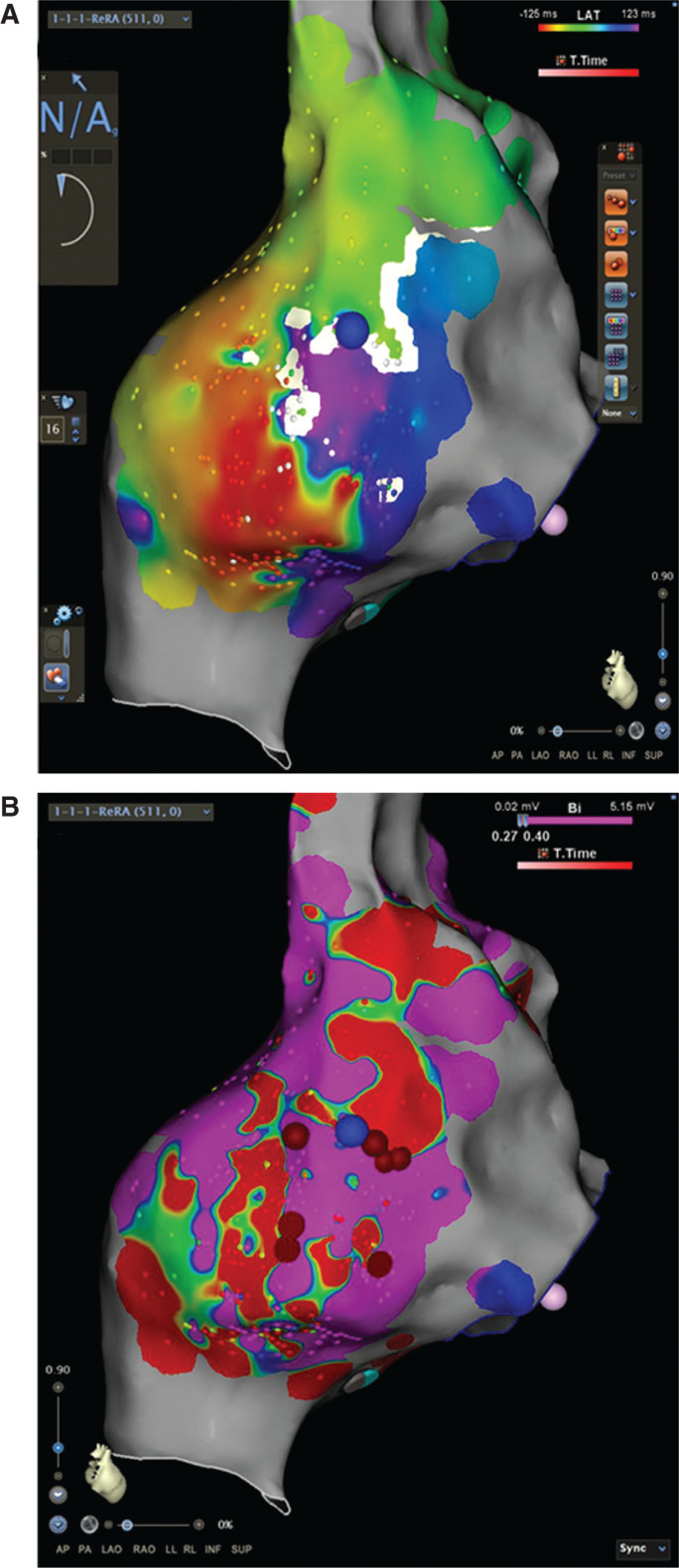
Electroanatomic mapping of SVT2. **A:** Activation map of SVT2, which used a zone of slow conduction in the posterolateral RA free wall. **B:** Voltage map of SVT2. Note that the voltage calipers have been adjusted with the lower limit set to less than 0.02 mV. With these settings, several conducting channels are identified coursing through the slow conduction zone indicated in the activation map. Red tags: ablation lesions; blue tag: the entrainment location, where the postpacing interval of the tachycardia cycle length was 21 ms. The RA is presented in a posteroanterior arrangement.

Following this, low-dose isoproterenol was initiated. Burst pacing was again performed, which induced SVT with a slightly different atrial activation sequence (tachycardia cycle length: 236 ms) and 1:1 AV conduction **(Figure 6)**. This tachycardia was hemodynamically unstable and required synchronized direct current cardioversion. The cavomitral isthmus was remapped during proximal CS pacing, which demonstrated persistent conduction block. Tachycardia was re-induced and persisted with 2:1 AV conduction. Activation mapping was again performed with the multipolar mapping catheter, which demonstrated a macro-reentrant atrial tachycardia originating from the RA free wall **(Figure 7)**.

With manipulation of the bipolar voltage calipers, several small channels were identified that corresponded to areas of slow conduction identified during activation mapping. Entrainment was performed in this region, yielding concealed fusion with a postpacing interval minus the tachycardia cycle length equal to 21 ms. RF application here resulted in tachycardia termination. A series of consolidation lesions were placed in areas where low bipolar voltage corresponded to arrhythmogenic channels identified in the propagation map. These were mainly located in areas of putative scar border zone and diastolic activity during tachycardia along the RA free wall. Afterward, extensive atrial burst pacing both during isoproterenol infusion and washout failed to induce any further SVT. She returned to the pediatric ICU without complications, recovering well. She was started on atenolol 25 mg once daily and discharged home the following day on her chronic medical regimen. She showed no subsequent SVT at six months of follow-up.

## Discussion

Patients with cc-TGA are rare, accounting for just 0.05% of all patients with CHD.^5^ Furthermore, the complex anatomic arrangement of conduction systems within the cc-TGA heart may lead to normal, anterior, or dual AV nodal systems, adding to variability in arrhythmia presentation.^6^ Our patient’s particular subset of associated congenital defects and surgical history makes her condition exceptionally rare.

The success of catheter ablation in adolescents and adults with CHD has improved significantly over time, likely related to advances in high-density mapping technology and ablation techniques.^7–10^ Of significant concern among all types of CHD is the recurrence of previously ablated or the appearance of new atrial arrhythmias. Newer mapping methods, including the use of high-density mapping, reduce the number of lesions (usually focal, rather than linear) required in ablation, which is likely to limit the likelihood of the creation of new arrhythmia substrate. Recurrence generally occurs within the first year after ablation (88%); those who do not experience a recurrence within this time period have a 91% chance of long-term cure.^11^

The overall prevalence of atrial tachycardia in patients with a transposition complex is 28%, although less is specifically known about the cc-TGA population.^12^ Reports of tachyarrhythmia treatment in the cc-TGA patient are sporadic, making it difficult to generalize about likely arrhythmia mechanisms.^13–17^ Studies focused upon atrial tachycardia in surgically repaired CHD do not typically carry sample sizes large enough to demonstrate isolated unique findings in the cc-TGA patient. In one study examining atrial tachycardia following the surgical repair of CHD, two of two patients with cc-TGA showed intra-atrial reentrant tachycardia (IART), whereas only three of six patients with dextro-transposition of the great arteries (d-TGA) displayed IART. This result may be linked to the particular type of procedure performed; while all six d-TGA patients underwent either a Mustard or Senning procedure, cc-TGA patients in this study received excision of subvalvular pulmonary stenosis, which possibly contributed to the arrhythmogenic substrate.^18^

In patients with biventricular physiology following surgical repair of complex congenital cardiac lesions, the two most important anatomic obstacles allowing for RA macro-reentry are the tricuspid annulus and right atriotomy.^19,20^ In general, typical atrial flutter using the cavotricuspid isthmus is the most common atrial arrhythmia mechanism in adult CHD patients,^20^ likely due to an increased degree of conduction delay through the crista terminalis and prolongation of atrial muscle refractoriness caused by atrial dilation and remodeling.^21–23^ While our patient’s systemic venous AV valve was a mitral valve (owing to the truism that “the valve goes with the ventricle”), ablation of the initial atrial tachycardia was successful by transecting the cavomitral isthmus, suggesting that the underlying arrhythmia substrate is likely similar to that of anatomically normal patients with typical atrial flutter. As such, typical differential pacing maneuvers can be adopted to confirm conduction block as was the case in our patient.

As seen in the present case, IART is the next most common atrial arrhythmia mechanism in patients with prior surgery for CHD.^24^ Importantly, the atrial activation sequence during tachycardia cannot reliably distinguish between tachycardia mechanisms unless multiple RA catheters are used. Thus, electroanatomic activation mapping and entrainment mapping are important tools for defining the arrhythmia mechanism as well as identifying critical components of the reentrant circuits that are amenable to ablation. In our patient, the initial tachycardia was confirmed to be a cavomitral isthmus–dependent flutter by activation and entrainment mapping.

Meanwhile, the second atrial tachycardia had a similar activation sequence but showed essentially vertical activation in the CS recordings. Typically, this is suggestive of a focal left atrial tachycardia, peri-mitral reentry, or roof-dependent flutter. However, as noted above, mapping and entrainment maneuvers identified this tachycardia as IART using the lateral atriotomy. There are several possible explanations for these disparate findings. In general, left atrial activation depends upon conduction over Bachmann’s bundle, through the interatrial septum, and through the CS musculature. In normal subjects, early activation is often noted in the distal CS due to Bachmann’s bundle connections near the base of the left atrial appendage. In this patient, slow conduction through the CS muscle and interatrial septum during IART may have allowed for a relatively greater contribution from Bachmann’s bundle to left atrial activation. Additionally, the tendon of Todaro and crista terminalis create natural barriers to conduction into the lower RA and, in the setting of altered intra-atrial conduction due to prior cardiac surgery, this may have preferentially directed activation through Bachmann’s bundle, leading to the resulting CS recordings seen in this case.

The arrhythmia substrate in IART likely relies on low-voltage “channels” coursing through the scar that allow for the slow conduction required for sustained reentry.^19,20,24^ Thus, voltage mapping can also be used to identify the critical circuit isthmus. The bipolar electrogram amplitude used to define scar varies: while many studies to date have defined scar as areas with a bipolar voltage of less than 0.1 mV, Nakagawa et al. only considered true scar as areas with no electrogram recorded above background noise, which can be as low as 0.03 mV. In the small study by Nakagawa et al., nearly half of successful ablation sites (7/15) were located in areas that would otherwise have been defined as “nonconducting scar” using a definition of bipolar voltage being less than 0.1 mV.^25^ Of obvious importance when using this approach is the assessment of the background noise present during mapping, which can vary significantly from laboratory to laboratory and which is affected by many external factors including cabling through the laboratory, proximity to alternating current power sources, and patient warming devices. In the present case, we were able to manipulate the voltage calipers in the mapping system to identify channels of relatively lower voltage coursing through the scar, which allowed us to create a complete line of block through the scar and prevent further SVT. Notably, using this approach, the absolute bipolar voltage below which the “scar” is annotated was less important than the relative voltage traversing between the putative slow-conduction channels and normal tissue. Channels identified in this manner can be compared to the activation map of the tachycardia to determine which channels may form critical circuit elements.

## Conclusion

We report a rare case of a cc-TGA patient with prior tricuspid valve replacement and RV outflow tract surgery in whom multiple atrial tachycardias causing syncope were successfully treated with catheter ablation. The combinatorial use of activation, entrainment, and voltage mapping may assist in the ablation of atrial arrhythmias in complex CHD, whereas high-density mapping can be used to augment the understanding of a tachycardia’s endocardial activation sequence and scar border zones. In the cc-TGA patient, an awareness of inherent anatomical nuances coupled with the potential for multiple tachycardia mechanisms is essential to periprocedural planning and successful mapping and ablation.
